# Some Drug Reactions in Urine

**Published:** 1908-10-24

**Authors:** 


### SOME DRUG REACTIONS IN URINE.

Various colourations and reactions of the urine may be due to something that the patient is taking by the mouth. The following is a brief summary of some of the commoner drug reactions in urines:

Copaiba, Copaiba resin, Cubebs, Balsam of Peru, Balsam of Tolu, and other resins and balsams all cause the urine to give a white ring when the nitric acid test for albumen is employed. The possibility of this source of fallacy leading to grave errors, in connection with life insurance, for example, is obvious. The white ring is due to the resin in the urine being precipitated by the nitric acid. The ring is less well defined, and less closely related to the line of junction between the urine and nitric acid than is that caused by albumen; and on adding a few drops of rectified spirit the coagulum of albumen does not dissolve, whereas a resin disappears at once.

Rhubarb and Senna contain chrysophanic acid, which gives the urine a bright yellow-brown colour. When suspected, the first test is to add an alkali, such as caustic soda solution or liquor potassæ, when the colour deepens to a red or red-brown. It is distinguished by two further tests: first, the red colour persists on standing instead of fading as some others do; secondly, the colour is removed from the solution and carried down in the precipitate on adding a barium salt, such as solution of barium chloride.

Santonin causes the urine to have a peculiar yellowish-green colour, due to the presence of oxysantonins. The addition of an alkali causes a red colour to appear, as in the case of rhubarb and senna, but this red is not precipitated by barium chloride; moreover, if the urine which has been alkalised is allowed to stand the red colour fades gradually away, which is not the case with that due to chrysophanic acid, rhubarb, or senna.

Salicylic acid, methyl-salicylic acid, mesotan, aspirin, aceto-salicylic acid, sodium salicylate, and other preparations containing the salicyl radicle cause the urine to give a deep reddish-brown or reddish-purple reaction with ferric chloride solution. The main importance of this lies in the fact that it may cause confusion when aceto-acetic (di-acetic) acid is being tested for in cases of diabetes mellitus, and so forth. For one thing, if di-acetic acid is present in a urine it is usual for acetone to be present also; acetone is readily recognised by the sodium nitroprusside test; if, therefore, in any given case the sodium nitroprusside test is negative, and yet the urine gives a strong ferric chloride reaction, some source of fallacy in the diacetic acid reaction would be suspected, such as the presence of salicylic acid products in the urine. There are two subsidiary points of distinction between the ferric chloride reaction, due to diacetic acid and that due to salicylates—namely, first, that the reddish-purple colour persists on boiling if it is due to salicylates, whereas it is discharged if due to diacetic acid; and, secondly, if the cold urine, to which ferric chloride has been added, is shaken up with ether, the red colour due to interaction of ferric chloride and diacetic acid is taken up by the ether and thus extracted, whereas the red substance produced by the interaction of ferric chloride and salicylates is insoluble in ether.

Antipyrin and drugs of the antipyrin series may be a source of considerable confusion in urine-testing in cases in which the patient is taking the drug unknown to others; at the same time, a knowledge of drug reactions in the urine may assist one in detecting the habitual secret taking of hypnotics such as antipyrine. The urine gives a red-brown or red-purple reaction with liquor ferri perchloridi, precisely similar to that caused by salicylic acid, and, like the latter, the reaction persists in spite of boiling, and the red substance is insoluble in ether. At the same time the urine is very likely to give some reduction of Fehling's solution without there being any crystals on applying the phenylhydrazine test, and without there being any fermentation of the urine by yeast.

Tannic acid, gallic acid, and tannigen cause the urine to go very dark on the addition of ferric chloride solution owing to the formation of ink by some of the tannates or gallates appearing in the urine.

Carbolic acid, creosote, and arbutin, the active principle of uva ursi, may cause the urine to assume a characteristic colour that may be anything from pale greenish to dark green, almost black. This colour may not be present when the specimen is first voided, but develops gradually from oxidation on exposure to the air. It is seldom that any difficulty of diagnosis arises, because the source of the carbolic acid is at once suspected and traced. Bile pigments may be excluded by the absence of jaundice in the patient, and by the fact that the urine does not give Gmelin's test. The urine of an alkaptonuric subject may present similar colouration, but in him the history would be a continuous one of long standing.

Potassium bromide causes a brown discolouration of the urine when nitric acid is added, owing to the liberation of free bromine. If a little chloroform is added, the bromine is extracted by the latter. The other substance which may give a similar reaction is indican, which sometimes gives a blue, sometimes a rose-red, colour on oxydation with nitric acid; in each case chloroform extracts the coloured substance, but the brown-red of bromine is quite unlike the rose-red of one of the indican products.

Potassium iodide and iodoform cause a reaction with nitric acid, by which iodine is liberated from the iodide in the solution. Moreover the tincture of guaiacum test for blood gives a greenish-blue reaction in the presence of iodides even when blood is absent. This behaviour of the guaiacum test in patients taking iodides is important to keep in mind.

The following drugs may cause the urine to reduce Fehling's solution: chloral, chloral hydrate, butyl chloral hydrate, carbolic acid, salicylic acid and its allies, camphor, and the antipyrine series.

Morphine, glycyrrhizin and the various preparations of liquorice, amyl nitrite, volatile oils, carbon monoxide, hydrocyanic acid, sulphuric acid, mercury salts, and lead may also produce an increased formation and elimination of glycuronates, and therefore some reduction of Fehling's solution. In all these cases, however, neither of the two confirmatory sugar tests—that by fermentation and that with phenylhydrazine hydrochloride—is positive if the reduction is due solely to the effects of the drug.

